# Measurement of Effects of Different Substrates on the Mechanical Properties of Submicron Titanium Nickel Shape Memory Alloy Thin Film Using the Bulge Test

**DOI:** 10.3390/mi12010085

**Published:** 2021-01-15

**Authors:** Nhat Minh Dang, Zhao-Ying Wang, Ti-Yuan Wu, Tra Anh Khoa Nguyen, Ming-Tzer Lin

**Affiliations:** 1 Graduate Institute of Precision Engineering, National Chung Hsing University Taichung, Taichung 40749, Taiwan; d108067005@mail.nchu.edu.tw (N.M.D.); g2001ya@yahoo.com.tw (Z.-Y.W.); paxon1992911@gmail.com (T.-Y.W.); khoa.eur@gmai.com (T.A.K.N.); 2 Center for Advanced Science Technology, National Chung Hsing University, Taichung 40749, Taiwan

**Keywords:** shape memory alloys, bulge test, Cr adhesion layer, residual stress

## Abstract

This study investigated the effects of different substrates on the mechanical properties of Ti-60at%Ni shape memory alloys (SMA). Two types of samples were prepared for this experiment: (1) a Ti-60at%Ni deposited on SiNx, and (2) a Ti-60at%Ni deposited on SiNx/Cr; both had a 600 nm thick film of Ti-60at%Ni. Deposition was done using the physical vapor deposition (PVD) process, and the microstructural changes and crystallization phase changes were observed through scanning electron microscopy (SEM) and X-ray diffraction (XRD). The results showed that the TiNi thin film with a Cr adhesion layer had better mechanical properties. The bulge test showed that TiNi thin film with a Cr adhesion had a higher Young’s modulus and lower residual stress. From the thermal cycling experiment, it was found that the Cr adhesion layer buffered the mismatch between TiNi and SiNx. Additionally, the thermal cycling test was also used to measure the thermal expansion coefficient of the films, and the fatigue test showed that the Cr layer significantly improved the fatigue resistance of the TiNi film.

## 1. Introduction

With the advancement of semiconductor technology, intelligent materials with actuation ability have become an essential component in the development of micro-electro-mechanical systems (MEMS). In particular, shape-memory alloys (SMAs) have several good properties that can restore or generate the phase-change stress-strain under hot or cold conditions. Among SMA films, TiNi films have been observed to be sensitive to environmental changes such as temperature and stress. Therefore, it is an ideal material for micro-sensor applications [1]. Although some micro-actuators for TiNi-based thin films have been developed [2,3], the factors affecting the phase transition of TiNi SMA films are not yet fully understood.

In particular, the impact of third-element adhesion materials is a point of interest. In order to enhance adhesion [4], very thin adhesion layers can be applied between the substrate and the metal films. The most used adhesion metals are Ti and Cr, however, the advantages and disadvantages of the individual materials as adhesion layers between TiNi SMA films and Si/SiNx substrate arepoorly understood. For example, Ti and Cr are commonly used as a simplified adhesion layer in the micro and nanofabrication field. They are known to be more chemically reactive than noble metals, and thereby increase the adhesion as they chemically bond to the substrate. However, the addition of a third element that could replace Ni and/or Ti has a substantial effect on the phase transformation behavior in TiNi alloys. As a result, the addition of Cr has attracted a lot of attention because it not only adjusts the transformation temperature [5], but also leads to the improvement of mechanical properties such as yield stress [6], torque capacity and rigidity [7], and fatigue life [8].

In this study, the properties of the different adhesion layers and how they affect the microstructure and crystallization phase, thus, influencing the mechanical properties of the sputtered TiNi film, are discussed. The differences in the mechanical properties of Ti-60at%Ni SMA deposited on SiNx and Ti-60at%Ni SMA deposited on SiNx/Cr after undergoing the bulge test were observed with an optical system. The trend in the stress evolution of different substrates was also examined through changes in temperature.

## 2. Method

### 2.1. Sample Fabrication Process

**Figure 1:**
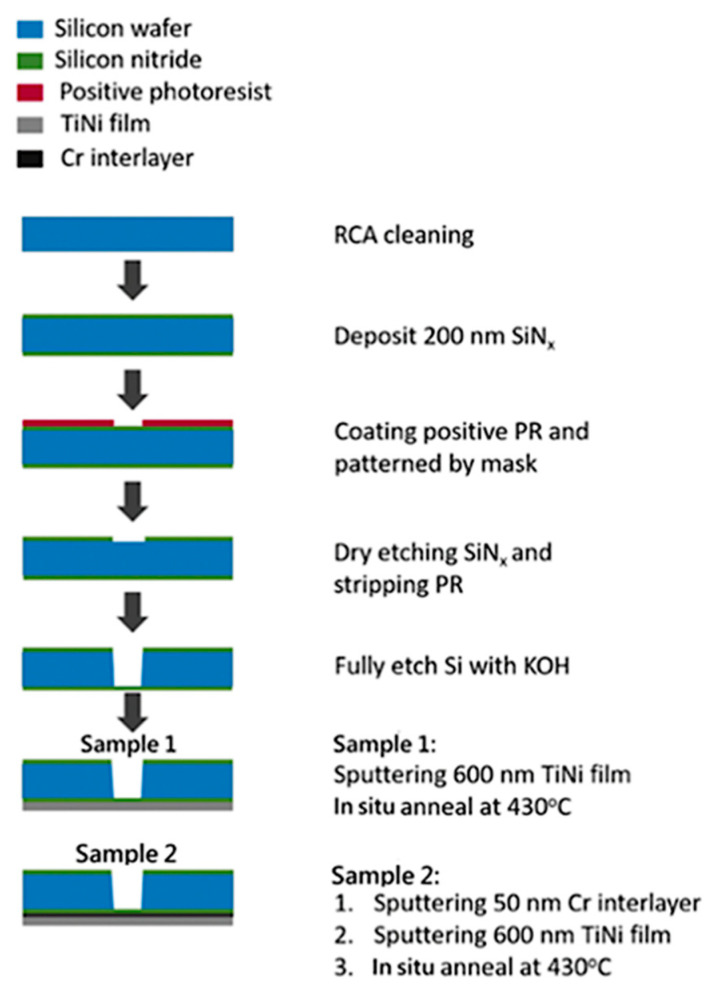
Experimental procedures for the bulge specimen.

**Figure 2:**
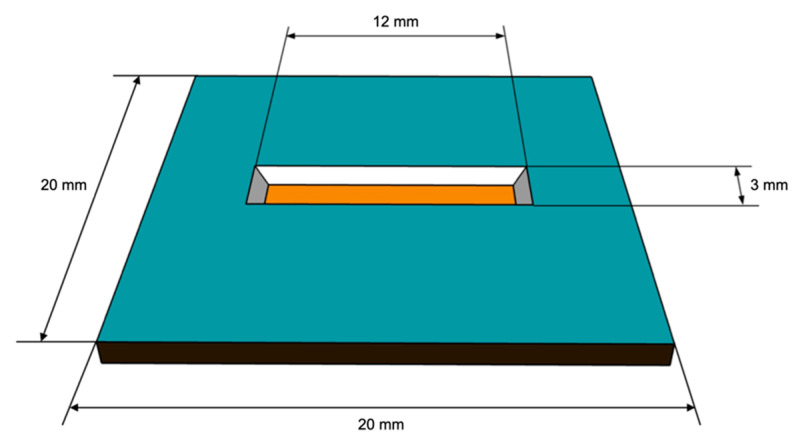
The dimensions and size of the bulge specimen.

As shown in Figure 1, the sample fabrication process has six steps. A <100>, 4 inches, double-sided polished 250 μm silicon wafer was used. The first step involved cleaning the wafer with RCA, which is commonly used to remove organic residues on silicon wafers, and depositing a 200 nm SiNx layer on the wafer through low pressure chemical vapor deposition (LPCVD). After deposition, the pattern was defined by lithography process technology; then, the wafer with the photoresist was placed in the inductively coupled plasma (ICP) for dry etching to remove the pattern not protected by the photoresist. Next, the SiNx layer was transformed into a floating membrane structure through wet etching. Finally, the bulge specimen shown in Figure 2 was produced by using the PVD process to deposit SMA thin films on top of the membrane. Two kinds of specimens were produced: one SMA deposited on SiNx only (Sample 1), and another SMA deposited on SiNx and a 50 nm Cr adhesion layer (Sample 2).

The test specimen is shown in Figure 2. It has a frame size of 20 mm × 20 mm and a thickness of 250 μm. After the sample fabrication, the 12 mm × 3 mm thin film membrane with a thickness of 800 nm can be found in the center and can be used for bulge testing.

The SMA effect only occurs in the crystalline state, however, the TiNi film sputtered at room temperature is amorphous, and thus it needs to be post annealed (usually at higher than 500 °C) to recrystallize it into a B2 phase structure. Previous studies have pointed out that if the film is deposited at a higher temperature (about 400 °C), this is sufficient to form a crystalline phase [9], so, in this experiment, a synchronous heat of 430 °C was used for annealing (in situ anneal) using the PVD system. The substrate temperature was maintained at 430 °C during the post-coating process to eliminate the need for subsequent high-temperature recrystallization annealing, which allowed the TiNi film to retain its crystalline state.

### 2.2. Bulge System

**Figure 3:**
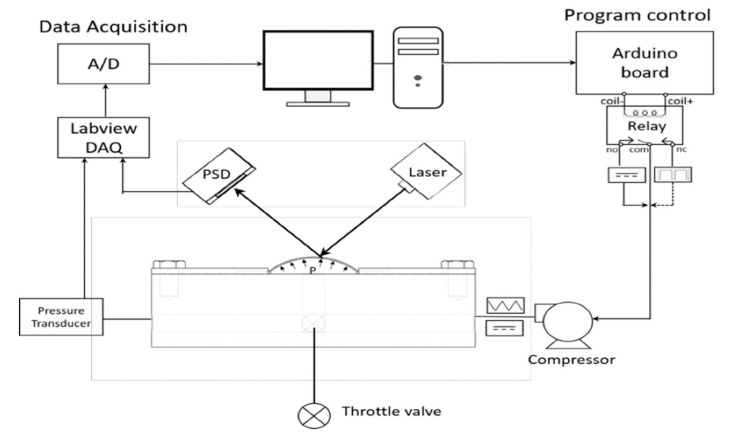
Diagram of the bulge system.

**Figure 4:**
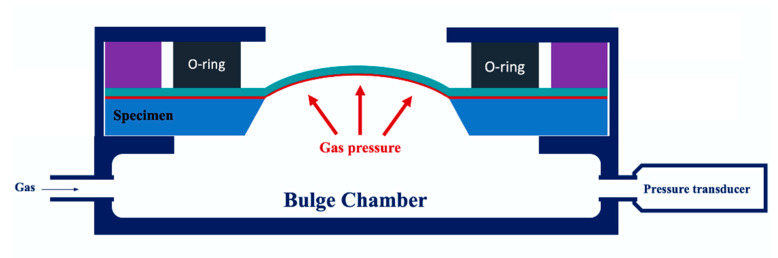
Schematic of the measuring system.

The construction of the bulge system is shown in the diagram in Figure 3. The experimental processes are illustrated in Figure 3. The system was mainly divided into a signal acquisition unit and an output control unit. The details of the bulge system setup can be found in our previous paper [10]. Figure 4 shows the details of how the bulge system was arranged with a pressure chamber. The bulge specimen was tightly clamped on top of the pressure chamber with O-ring fasteners. After the sample was sealed, the gas was pumped into the pressure chamber to provide the pressure for the bulge membrane. The bulge height was measured with a position sensing detector (PSD). The signal acquisition unit was received primarily by the NI-Data Acquisition (DAQ) box through a pressure transducer and a PSD, which returns the voltage signal. The control unit used the Arduino single-chip controller to regulate the electromagnetic relay using Arduino’s integrated development environment (IDE) open-source program. By changing the voltage signal in the air compressor, it was able to produce different pressure modes.

### 2.3. Experimental Procedures

In order to investigate the effects of two different substrates on the mechanical properties and crystal structure of the TiNi SMA thin films, we utilized the films on pure silicon nitride film and silicon nitride coated with a chromium adhesion layer.

Three different type of tests were performed using the bulge test and several samples of each kind were tested. First, each sample underwent the standard pressure-bulge loading/unloading monotonic test to obtain the pressure-displacement curve, which was converted into the stress-strain relationship. Tests were performed repeatedly on several randomly selected groups of specimens from the same batch of specimens to obtain the stress-strain relationship diagram. This provided the original residual stress and the Young’s modulus of the tested films. After the linear pressure-bulge loading/unloading monotonic test, the sample then underwent thermal cycling experiments or the fatigue tests. 

For the thermal cycling experiment, the samples underwent a standard heating/cooling test, which went from room temperature to 80 °C without pressure loading. This thermal cycle test was used to measure the thermal expansion coefficient of the films and study the thermal stress evolution of thin films during heating/cooling conditions.

For the fatigue cycle experiments, each of the samples was tested using a standard pressure-bulge loading/unloading monotonic test before a set number of fatigue cycles.

Before the fatigue test, a monotonic loading test was performed on several randomly selected groups of specimens from the same batch of specimens to obtain the stress-strain relationship diagram. In applications of SMA thin films, the average stress level of their life cycles is mostly within the elastic range of the specimen. Therefore, the measurement range of stress on specimens for its loading/unloading monotonic and fatigue tests were performed based on these.

**Figure 5:**
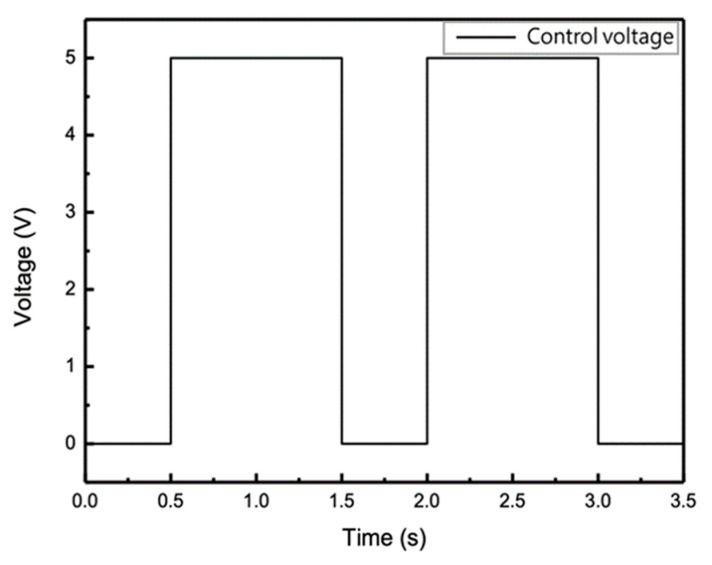
Pulse voltage signal output by Arduino Uno.

**Figure 6:**
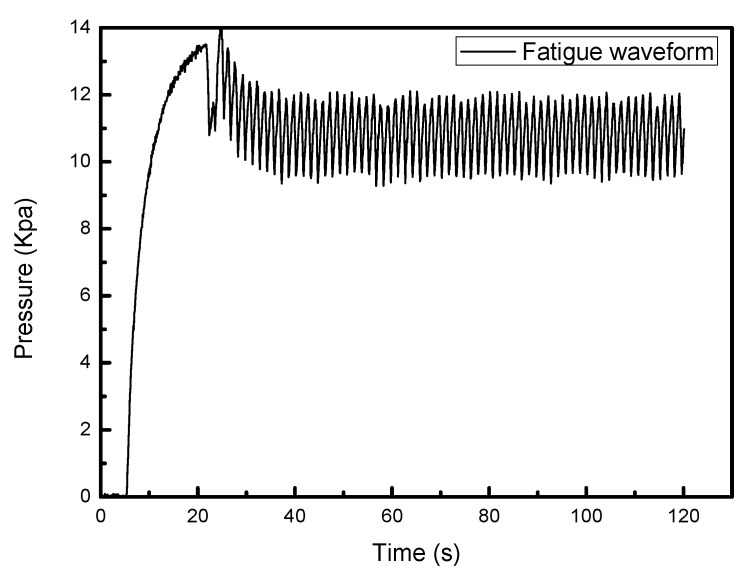
Fatigue test waveform output by air compressor.

During the fatigue test, we used Arduino Uno software (Board Model UNO R3, Arduino IDE 1.8.13, Ivrea, Italy) to control the compressor, this provided the initial setting of a constant pressure of 2.5 KPa for the bulge specimen to provide a mean stress of 300 MPa, and then the Arduino IDE program was used to control the relay switch of a pulse signal with a frequency of 0.67 Hz by changing the voltage signal in the air compressor; therefore, this was able to produce a different stress amplitude of 25 MPa from the pressure modes as shown in Figure 5. The fatigue program produced an air compressor output with a triangular waveform and a pressure range of 9.5~12 KPa, as shown in Figure 6 and stress after conversion was equivalent to 300 ± 25 MPa. In order to study the material properties of the samples before and after the fatigue tests, all the samples were set to be tested at fatigue cycles of 5 × 10^4^ to maintain the sample’s integrity before its fatigue limit for further study.

After a set number of fatigue cycles, the sample was tested with the standard pressure-bulge loading/unloading monotonic test and comprehensive microscopic observation.

**Figure 7:**
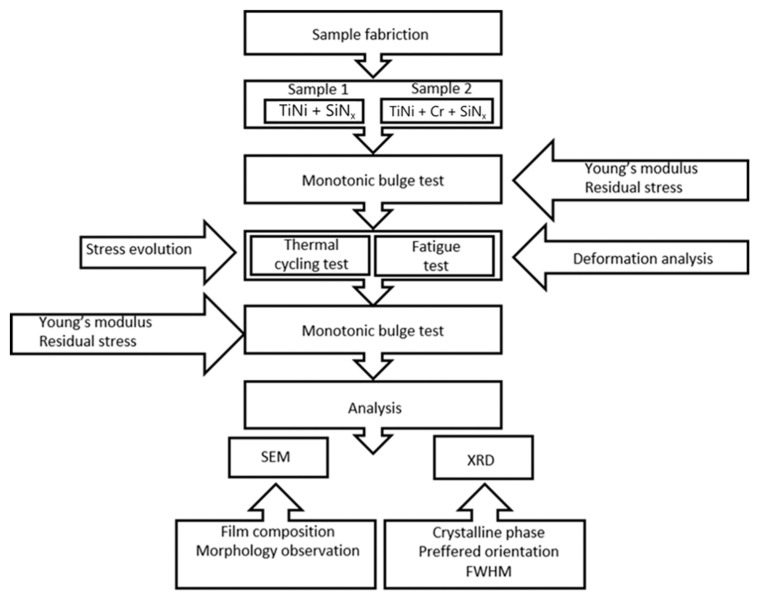
Schematic diagram of the experimental procedure.

The schematic diagram for this experiment is shown in Figure 7.

## 3. Results and Discussion

### 3.1. Microstructure and Surface Topography of the Specimens

In this section, the effects of different substrates on the microstructure and surface topography of two types of specimens (Specimen 1 and 2) under the same coating condition are presented and discussed.

#### 3.1.1. Surface of the Specimens by FESEM

**Figure 8:**
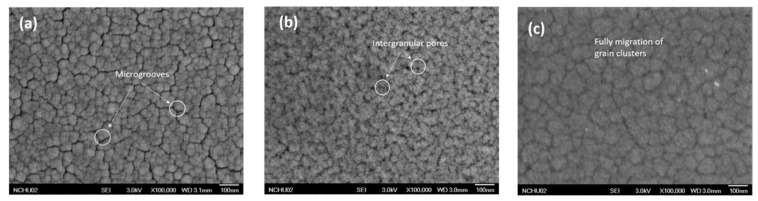
Surface of TiNi thin film: (**a**) Unannealed; (**b**) Specimen 1; (**c**) Specimen 2.

Figure 8 shows that the unannealed specimen had a low atomic kinetic energy and was less migrated after deposition on the surface of the substrate; thus, it exhibited island-like particle clusters with clear boundaries between each cluster. As clearly presented in Figure 8b,c, there are numerous trenches on the TiNi thin film surface, the brittle-hard TiO_2_ oxide layer formed due to the intense stress evolution, and the size and distribution of the wrinkle of the crystalline film were significantly different from the unannealed film.

The coalescence can be clearly observed in the annealed crystallized specimens, and the amorphous TiNi films have a smaller granular structure. As can be seen in Figure 8b,c, where the film was annealed at 430 °C, the adsorbed atoms are pulled toward the micropores since the particles have large kinetic energy when the substrate is deposited.

#### 3.1.2. Cross-Sections of the Specimens by SEM

**Figure 9:**
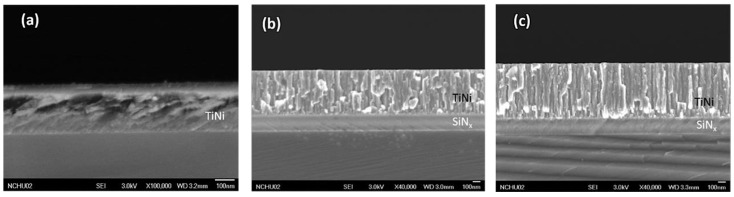
Cross-sections of the films obtained through SEM: (**a**) Unannealed; (**b**) Specimen 1; (**c**) Specimen 2.

Figure 9 presents the cross-sections of the films, showing the TiNi upper layer and the SiNx lower layer. It can be seen that the unannealed TiNi film (Figure 9a) had no obvious change in microstructure, and that Specimens 1 (Figure 9b) and 2 (Figure 9c) had clusters of columnar crystals between the layers of the entire film. Additionally, the film containing the Cr bonding layer (Specimen 2) had a more uniform structure than Specimen 1. This is probably because Specimen 2 achieved better migration in the growth process, allowing the particles to grow and merge uniformly; thus, the stratification of Cr and TiNi cannot be clearly seen. Moreover, it can be speculated that the Cr might have been diffused into the interior of TiNi during heating.

#### 3.1.3. Surface Composition Analysis by EDS

**Figure 10:**
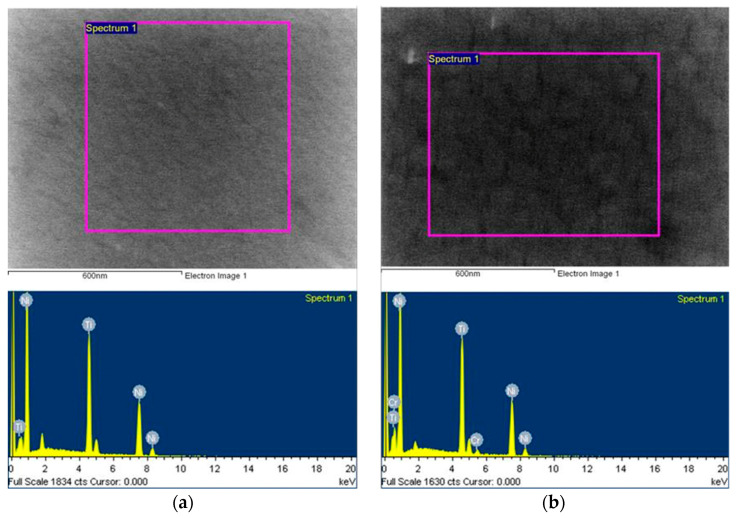
Surface composition analysis by EDS: (**a**) Specimen 1; (**b**) Specimen 2.

The surface analysis results from the EDS are shown in Figure 10a,b and the images were taken immediately after the specimens were taken out of the PVD vacuum chamber. All of the tested NiTi specimens were deposited and annealed to form an SMA crystallographic structure inside the chamber under vacuum whereby Argon gas was purged during the deposition and annealing processes. The formation of TiO_2_ during the PVD and annealing processes becomes very limited although previous literature has shown that i a thin layer of TiO_2_ can be easily formed on NiTi films [11]. According to the surface analysis by EDS, the atomic ratio of Ti to Ni in Specimen 1 was about 40:60 (see Figure 10), and that of Ti to Ni to Cr in Specimen 2 was 40:58:2, indicating that the ratio of TiNi in Specimen 2 was higher than in Specimen 1. The results are shown in Table 1.

#### 3.1.4. Profile Composition Analysis

**Figure 11:**
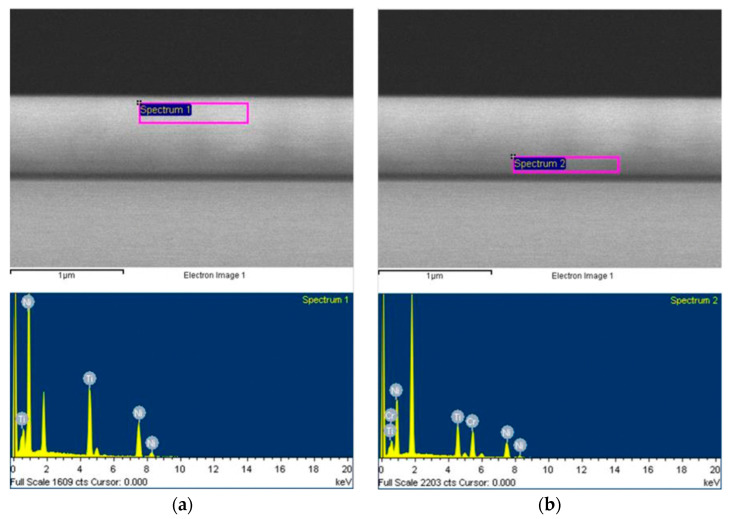
Cross-sectional composition analysis by EDS: (**a**) Surface of TiNi; (**b**) Middle layer of Cr.

**Figure 12:**
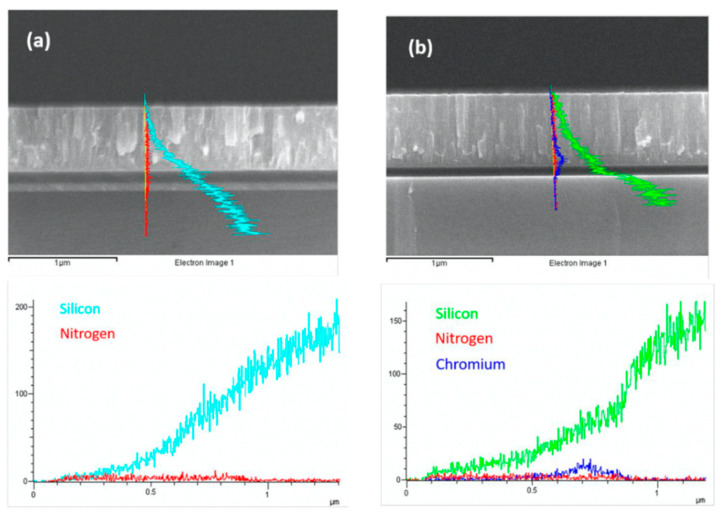
EDS line scan: (**a**) Specimen 1; (**b**) Specimen 2.

As can be seen from the profile analysis of Specimen 2 in Figure 11, Cr did not diffuse into the surface and was mainly concentrated at the bottom boundary. The results are shown in Table 2. As for TiNi, the surface atoms were higher and the bottom boundary was lower. With regard to the surface composition analysis of TiNi, which is shown in Figure 12, since the atomic radius of Ni and Cr are close to each other, the difference from Ti was large. According to the Hume-Rothery rule, when the difference in diameter between the two metals is less than 15%, there will be mutual solubility. These criteria are called the size factor [12], which allow substitutional solid solutions to form easily.

As shown in Figure 12, the SiNx layer blocked most of the Si diffusion, and the specimen containing the Cr intermediate layer had a lower content of Si and N; therefore, it can be inferred that the blocking diffusion effect is better in SiNx only. The barrier layer prevented Ti, Ni, and Si from forming a brittle compound with N.

### 3.2. Crystallization Phase Analysis of TiNi Films on Different Substrates

**Figure 13:**
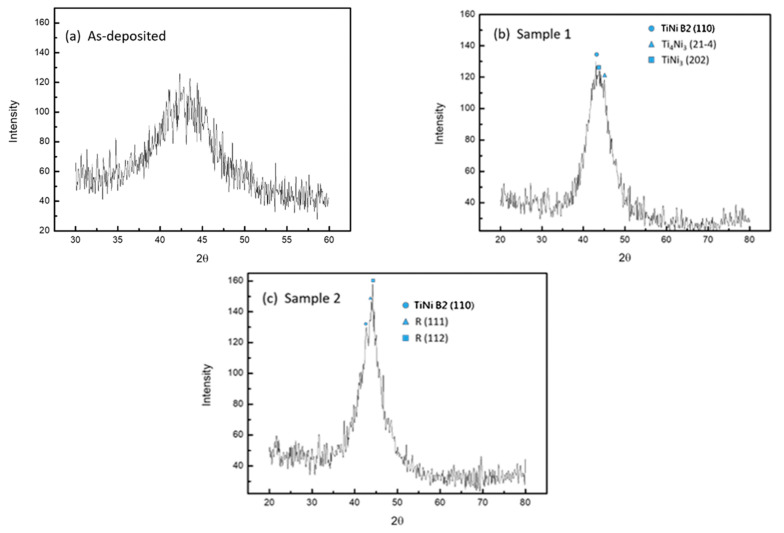
X-ray diffraction pattern for different coating conditions: (**a**) Unannealed; (**b**) Specimen 1 heated at 430 °C; (**c**) Specimen 2 heated at 430 °C.

Figure 13 shows the XRD phase diagram of the unannealed specimen, Specimen 1, and Specimen 2 at room temperature. The crystallization of TiNi was assessed based on the generation of the diffraction peak.

The reaction phase and full width at half maximum (FWHM) produced by the annealing process were analyzed by JCPDS card number and X’Pert High Score Plus software(Malvern Instruments Ltd., Worcestershire, UK).

#### 3.2.1. Crystallographic Analysis of the Unannealed TiNi Specimen

In the unannealed specimen phase diagram (see Figure 13a), it can be seen that there was a broad diffraction peak around 2θ of about 42°, which is the XRD characteristic of the amorphous TiNi. Therefore, SMA was not present in the unannealed TiNi specimen at normal temperature.

#### 3.2.2. Crystallographic Analysis of Specimen 1

The coated TiNi specimen heated at 430 °C had a significant diffraction peak around 2θ of about 42.6°, indicating that the film was crystallized, and that the crystal structure of the Ti_40_Ni_60_ film was complete. The crystal structure corresponding to this angle had a lattice constant of approximately 2.99 Å cubic crystal, corresponding to the (110) crystallographic direction of the B2 austenite phase. It can be judged that it mainly retained the high temperature austenite at normal temperature, indicating that there should be no shape memory effect caused by the change in the phase of the granules in the field during the heating process. Miyazaki et al. [13] found that the annealed crystalline TiNi film usually has a strong structure of austenite B2 phase (110). At room temperature, the (200) and (022) peaks of martensite are dominant. In addition, many diffraction peaks were found at 2θ of about 44° in the vicinity, which could be a precipitation phase such as Ti_3_Ni_4_ and TiNi_3_. This indicates that the Ni content of the film might be high, thus resulting in B2 of TiNi as the first crystal phase. Furthermore, the appearance of Ti-Ni-Si, the ternary phase of Ti-Si-N and Ni-Si-N, and the binary phases of Ti-Si and Ni-Si were not found, indicating that the SiNx layer had a good diffusion barrier effect.

#### 3.2.3. Crystallographic Analysis of Specimen 2

Ti_40_Ni_58_Cr_2_ was shown to be a crystal of B2 austenite + R phase at room temperature, and a hexagonal crystal structure was observed in the R phase, which is commonly found in TiNi alloys dissolved in a third element. Previous research by Bricknell et al. [14] showed that the crystallinity of TiNiCu alloy is mainly affected by several factors such as atom size, relative ion size, electronegativity, and state density at the Fermi level. This phenomenon can also be applied to the Ti_40_Ni_58_Cr_2_ alloy of this experiment. The literature [14] has demonstrated that the atomic sizes of Cr and Ni are similar but smaller than Ti, and the electronegativity of Cr is smaller than Ni but larger than Ti. Therefore, Cr will replace the position of Ni in Ti-Ni-Cr SMAs instead of Ti. Further, the present study considered that when the R phase was introduced, the mother phase had a hardening effect, so that slippage of the poor row became difficult; therefore, most of the generation of the mesophase had superior properties compared to the B19’ phase. In addition, during the diffraction, there was no precipitation of Ti_3_Ni_4_ and TiNi_3_ observed, which indicates that the composition of Specimen 2 is relatively uniform.

#### 3.2.4. Analysis of Crystallinity and Grain Size

The XRD diffraction pattern obtained in this experiment was mainly a single main peak. Therefore, the half-height width was obtained by fitting the diffraction peak of XRD with a Gauss curve. The grain information shown in Table 3 was obtained using the Sherrer equation.

Table 3 shows that the crystal grain size of Specimen 1 was small. Based on the result of the SEM surface morphology, it was found that Specimen 2 had a small half-height width, indicating that crystallinity is possible. In the study by Chen and Park [15], it was shown that the crystallization ability of an amorphous alloy depends on the stability of the alloy in the amorphous state. This stability can be defined by the enthalpy of mixing between the alloy atoms. If the mixed enthalpy is negative, the alloy forms an amorphous phase [16], which gives the alloy a stable phase in the amorphous state. Consequently, the study of Miedema [17] found that there is a large negative mixing enthalpy between TiNi atoms.

### 3.3. Measurement of the Mechanical Properties by the Bulge Test

The Young’s modulus and residual stress of Specimens 1 and 2 using the bulge system are discussed in this section, as well as the effects of the substrate on the mechanical properties of the TiNi films.

#### 3.3.1. Loading/Unloading Monotonic Test

**Figure 14:**
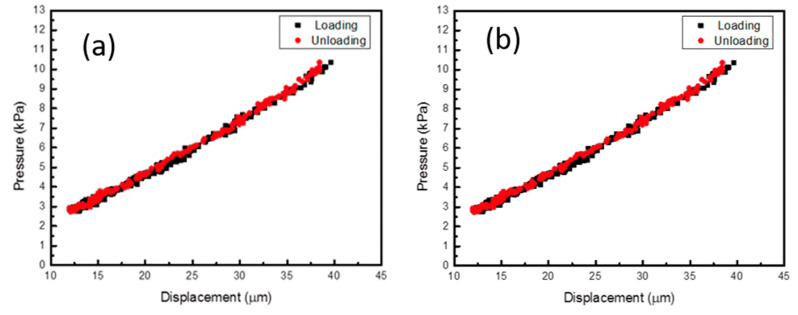
Loading/unloading displacement-pressure diagram: (**a**) Specimen 1; (**b**) Specimen 2.

Figure 14 shows the pressure-displacement plot of the loading/unloading monotonic test for each sample. This provided the original residual stress and Young’s modulus of the tested films. In order to maintain the integrity of the samples for further thermal cycling or fatigue tests, the loading/unloading monotonic test was only performed in the elastic region.

#### 3.3.2. Stress-Strain Relation of Monotonic Tests

**Figure 15:**
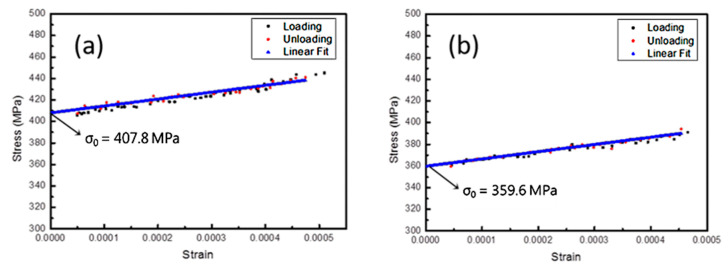
Mechanical property measurement by bulge: (**a**) Specimen 1; (**b**) Specimen 2.

Figure 15 shows the stress-strain relation of samples after the monotonic tests, which were converted from the pressure-displacement data from the loading/unloading test. Table 4 shows that the Young’s modulus of Specimen 2 was high and the residual stress value was relatively low. This indicates that at room temperature, the specimen containing the Cr adhesion layer had better mechanical properties than the TiNi film alone that was simply plated on the SiNx barrier layer. The strain can be calculated by Equation (1):

(1) ε=2H23(D2)2

where *H* is the height of the bulged thin films and *D* is the width of the rectangle membrane (3 mm). The stress *σ_xx_* of the film from the pressure-displacement data recorded during the experiment can then be calculated by:

(2) $$\sigma_{xx}=\frac{PR}{t}$$

where *t* is the film thickness, *P* is the applied pressure and *R* is the bulge radius of curvature.

#### 3.3.3. Residual Stress Evaluation

In this experiment, the coating was simultaneously heated at 430 °C. The thermal expansion coefficients (CTE) of TiNi and SiNx were 11.2 × 10^−6^/°C and 3 × 10^−6^/°C [18], demonstrating that the coefficients of thermal expansion differed greatly. According to the thermal stress Equation (1), when the film is cooled from an unconstrained state to a normal temperature, the amount of shrinkage of SiNx is small, and TiNi is subjected to tensile thermal stress of about 230 MPa. In addition, since the SiNx structure was trigonal (a = 7.766 Å, c = 5.615 Å) [19], which has a large lattice constant and a crystal structure different from the mother crystal of TiNi (a = 2.99 Å), it resulted in a misfit strain in the film growth stage, so, an internal stress of more than 400 MPa was left after the coating was completed. The coefficient of thermal expansion of Cr (6.20 × 10^−6^/°C) was between TiNi and SiNx, and the crystal structure (cubic, a = 0.2885 Å) was similar to TiNi, thus it was a very suitable intermediate buffer layer. Also, the results showed that the residual stress of the TiNi film containing the Cr intermediate layer was reduced by 48.2 MPa; this is considerably lower than that of the purely plated SiNx substrate. Therefore, the Cr intermediate layer is effective in decreasing the accumulation of thermal stress during annealing and cooling.

### 3.4. Evolution of Thermal Stress by Thermal Cycling Test

**Figure 16:**
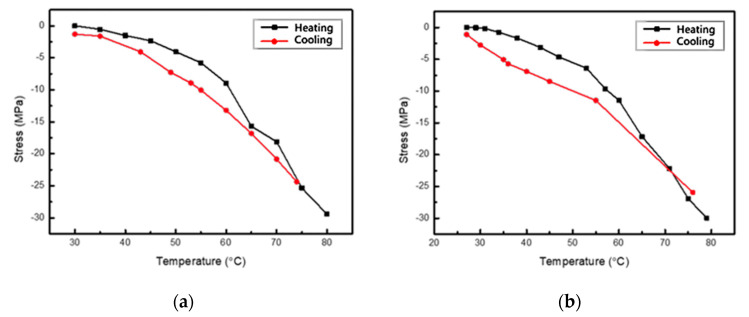
Temperature-stress evolution diagram of TiNi film: (**a**) Specimen 1; (**b**) Specimen 2.

In this experiment, there was no phase change observed in the TiNi film during temperature change. Also, only the thermal stress that did not match the SiNx substrate was considered. The stress-temperature curve is shown in Figure 16.

As can be seen in Figure 16, the film did not undergo phase change. As the temperature increased, the stress decreased, and the stress increased from low to high during cooling. Both the heating and cooling curves were close to a straight line and the slopes were all negative; the flatness of the line indicates that thermal stress is dependent on temperature.

(3) $$\varepsilon_{m}=\left(\alpha_{s}-\alpha_{f}\right)\left(T-T_{o}\right)=\left(\alpha_{s}-\alpha_{f}\right)\Delta T$$

(4) $$\sigma_{m}={\;E}_{f}\varepsilon_{m}=E_{f}\left(\alpha_{s}-\alpha_{f}\right)\Delta T$$

Since there was no phase change in the film when the temperature changed, the deposited film’s thermal expansion coefficient was evaluated using the above Equations (3) and (4), where $\alpha_{s}$, $\alpha_{f}$ are the TiNi substrate thermal expansion coefficient and $T_{o}$ is the initial temperature and $E_{f}$ is the film’s Young’s modulus. The obtained stress rate, the measured TiNi Young’s modulus, and the SiNx thermal expansion coefficient were substituted to calculate the thermal expansion coefficient. The results are shown in Table 5.

The tensile thermal stress in the TiNi film of Specimen 1 evolved at a higher rate and its thermal expansion coefficient was closer to the value obtained from previous literature [20]. Meanwhile, Specimen 2 containing the Cr bonding layer obtained lower values due to the Cr intermediate layer. The coefficient of thermal expansion was between TiNi and SiNx; therefore, it has a buffering effect that absorbs part of the thermal strain.

### 3.5. Material Properties of Samples after Fatigue Cycles

#### 3.5.1. Mechanical Properties after Fatigue Cycles

**Figure 17:**
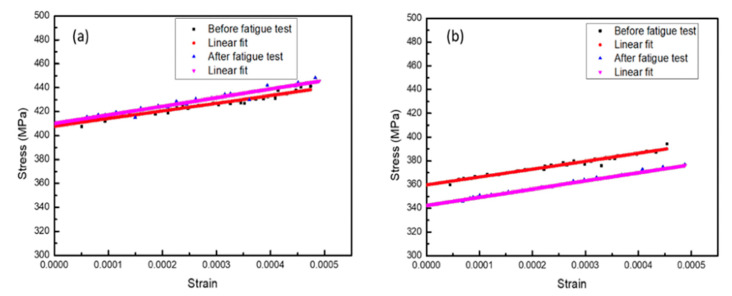
Change in mechanical properties before and after the fatigue test: (**a**) Specimen 1; (**b**) Specimen 2.

Figure 17 shows the bulge test stress-strain curve differences between Specimen 1 and Specimen 2 before and after 5 × 10^4^ fatigue cycles. The relevant data is shown in Table 6.

The residual stress was measured after Specimen 1 was subjected to fatigue effects, leading to anelastic strain. The Young’s modulus of Specimen 2 only increased slightly but there was a large release of residual stress.

#### 3.5.2. Ratcheting Behavior

**Figure 18:**
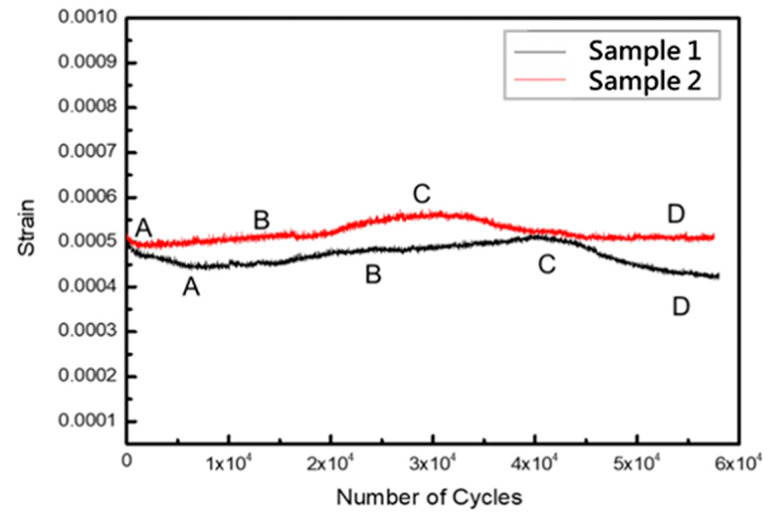
Curve of ratchet strain-cycle times for different specimens.

During the tests, the membrane was bulged up for loading and down for unloading. Therefore, the axial strain on the long side of the rectangular window can be regarded as zero, the short side at both ends can be assumed to be free-end, and the film is only subject to the uniaxial tensile force of the long side, so, only the measurement on the axis strain ($\varepsilon_{zz}$) can be measured. The $\varepsilon_{zz}$ ratchet strain of cyclic load is depicted in Figure 18, from which it can be seen that the first several cycles (A) had rapid hardening, which gradually softened to a stable state (B) after reaching a peak. In this stage, the increase in dislocation density and entanglement reduced the dislocation motion ability, demonstrating the hardening phenomenon. Among them, Specimen 1 without a Cr adhesion layer went through more cycles before reaching a stable state and there was an apparent softening phenomenon in Specimen 2. After several cycles, it started to produce a hardening phenomenon (C). Moreover, Specimen 1 showed severe hardening while Specimen 2 showed no noticeable change (D).

Although fatigue tests dynamic stress fell within the elastic region, only small dynamic stress produced plastic flow in each cycle, and the cycle creep [21] occurred due to the Bauschinger effect. Moreover, stress relaxation occurred along with microscopic deformations. This stress relaxation was caused by the difference in dislocation density and the decrease in storage strain energy [22,23]. The work hardening rate of Specimen 1 was relatively large, so the cyclic strain vibration was relatively small and stress relaxation was slow. On the contrary, Specimen 2 had a single primary slip system, thus its residual stress could release easily.

#### 3.5.3. Changes in FWHM and Diffraction Peak after Fatigue Testing

**Figure 19:**
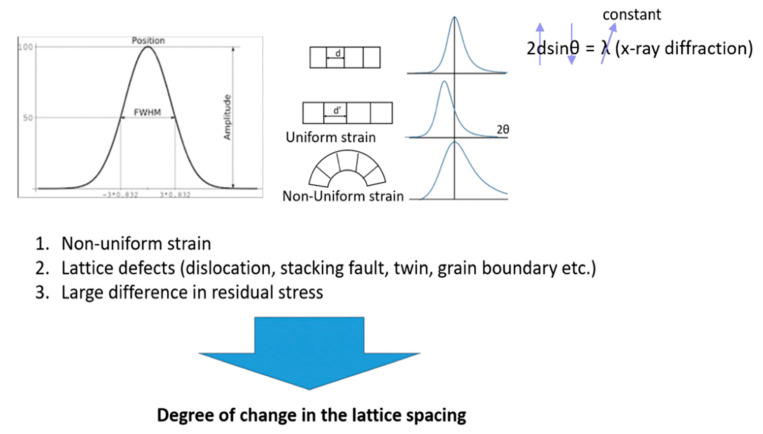
Schematic diagram of X-ray diffraction peak and FWHM.

According to Bragg’s law, the X-ray diffraction peak varies with changes in lattice spacing (shown in Figure 19).

**Figure 20:**
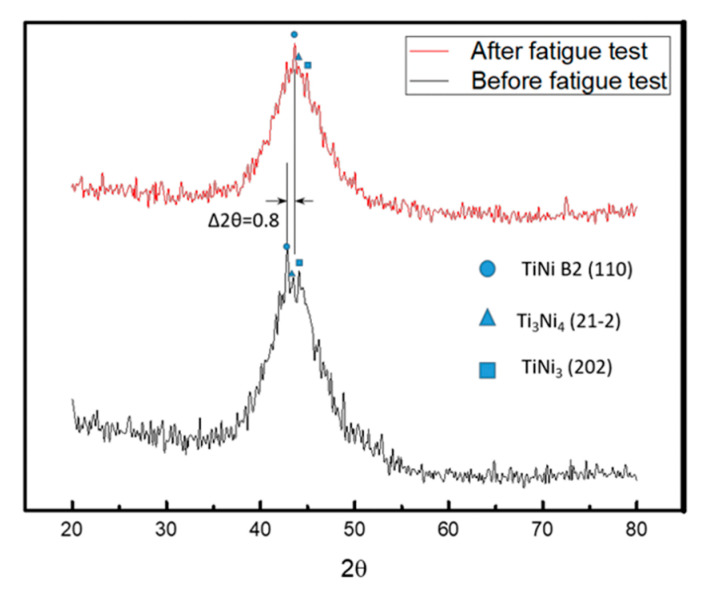
Diffraction peaks for Specimen 1 before and after fatigue testing.

The experimental data for all the specimens are shown in Figure 20 and Table 7. Based on the data obtained, Specimen 1 produced strain hardening. Thus, a great amount of dislocation and defect accumulated inside, which increased the density of dislocation. Consequently, it can be clearly seen that the FWHM became narrow. Also, the main peak of the diffraction peaks shifted to the right by a large angle, and the peak of the phase diagram was uniform. It is possible that the lattice spacing was reduced and the preferred orientation was generated due to the uniform strain of the lattice, indicating that Specimen 1 has reached fatigue in the third stage.

**Figure 21:**
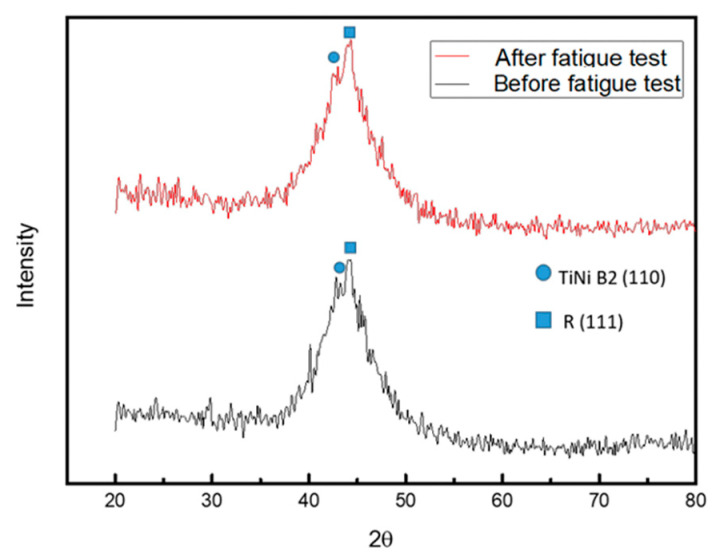
Diffraction peaks for Specimen 2 before and after fatigue testing.

As can be seen in Figure 21 and Table 8, the FWHM of Specimen 2 increased slightly. Besides, the position of the main peak did not change significantly, and a great amount of residual stress was released. Consequently, it was speculated that this occurred only during the first stage; the crack was initiated but did not reach the propagation stage. The presence of a solid solution of alloy elements in the material introduced an elastic stress field that reduced the strain energy of the surrounding lattice and the grain boundary, forming a dislocation partition that prevented the movement of the grain boundary. Therefore, it can be inferred that Specimen 2 contains solid solution alloy elements with better fatigue resistance.

## 4. Conclusions

This study examined the effect of different substrates on TiNi films and observed their influence on the films’ microstructure through analyzing the surface morphology and metallographic diagrams, and through measuring the Young’s modulus and residual stress using the bulge system. Finally, an experiment to study the evolution of thermal stress was performed to investigate the relationship between TiNi and the substrate in terms of thermal stress.

When coating TiNi, wrinkles on the surface of the brittle-hard TiO2 oxide layer can form due to intense stress evolution, and this can expand into cracks. After comparison, it was found that the TiNi film with a Cr-plated intermediate layer had fewer intergranular openings and grooves, indicating that the Cr layer can reduce the stress in the coating without causing shrinkage in the surface TiO_2_. Further, it was observed from the cross-section of the films that the TiNi with the Cr-bonded layer had better mobility and exhibited a uniform columnar crystal structure. This means that the Cr intermediate layer can effectively block the diffusion of Si and N to the TiNi film to form a brittle phase.

Moreover, the X-ray diffraction pattern showed that the TiNi directly plated on SiNx was mainly B2 Austenite, but due to the composition of Ni, precipitates such as TiNi3 and Ti_3_Ni_4_ were formed, which may have caused the uniformity inside the film and possibly affected its properties. Meanwhile, the TiNi plated on the Cr intermediate layer mainly showed the R phase structure of the rhombohedral crystal. This was the intermediate phase generated by the diffusion of the Cr element, which also retained a large number of B2 parent phase. Based on the FWHM analysis, the TiNi containing a Cr layer had better crystallinity.

The results of the single bulge test demonstrated that the TiNi with the Cr layer had higher elastic modulus. The thermal expansion coefficient and lattice constant of Cr were also found to be closer to TiNi; therefore, it can be regarded as a buffer layer between TiNi and SiNx. This buffer layer could potentially prevent excessive thermal and internal stresses caused by poor thermal expansion and lattice mismatch. Additionally, it was found that the residual stress value measured in the film with the Cr layer was lower than that of the Cr-free layer by about 48 MPa.

It was observed from the temperature evolution experiment that the temperature of the TiNi film rose to 80 °C from normal, and the resulting stress curve was negative and linear. This is probably because the thermal expansion coefficient of TiNi is larger than that of SiNx, so the substrate was subjected to high to normal temperature coating. Although constraint and extremely hot tensile stress were generated, the film was able to gradually recover as the temperature increased, leading to the gradual decrease of the stress value.

Comparing the two specimens, it was found that the stress rate of the film with the Cr intermediate layer was lower and the residual strain was smaller. It is presumed that the thermal expansion coefficient of Cr was between the two layers, and serves as a buffer that prevents deformation. In addition, in the temperature change test, it was found that the thermal expansion coefficient of an unknown film can be estimated from the substrate with known parameters using the bulge method.

## Figures and Tables

**Table 1:** Composition of the specimens.

(a) Sample 1		
Element	Weight%	Atomic%
Ti K	36.00	40.80
Ni K	64.00	59.20
Total	100.00	100.00
**(b) Sample 2**		
**Element**	**Weight%**	**Atomic%**
Ti K	35.50	40.19
Cr K	2.04	2.13
Ni K	62.46	57.68
Totals	100.00	100.00

**Table 2:** Composition of the specimens.

(a) Sample 1		
Element	Weight%	Atomic%
Ti K	36.86	41.71
Ni K	63.14	58.29
Totals	100.00	100.00
**(b) Sample 2**		
**Element**	**Weight%**	**Atomic%**
Ti K	23.32	26.15
Cr K	31.13	32.16
Ni K	45.55	41.68
Totals	100.00	100.00

**Table 3:** Size of grain.

Number	FWHM	Size of Grain (mm)
1	7.146	12.51
2	6.518	13.78

**Table 4:** Measurement of mechanical properties t of TiNi film.

Number	Composite Film Young’s Modulus	Composite Film Residual Modulus	Young’s Modulus of TiNi Film	Residual Stress of TiNi Film
1	102.8 GPa	310 MPa	64.1 GPa	407.8 MPa
2	102.0 GPa	279 MPa	66.8 GPa	359.6 MPa

**Table 5:** Parameter of thermal expansion.

Number	Average Stress Rate (MPa/°C)	Thermal Expansion Coefficient (1/°C)
1	−0.532	11.42 × 10^−6^
2	−0.481	10.04 × 10^−6^

**Table 6:** Mechanical parameters before and after fatigue testing.

	Before Testing		After Testing	
Number	Young’s Modulus (GPa)	Residual Stress (MPa)	Young’s Modulus (GPa)	Residual Stress (MPa)
1	64.1	407.8	71.9	410.0
2	66.8	359.6	68.9	324.4

**Table 7:** X-ray diffraction data before and after fatigue testing.

Fatigue Test	FWHM	Peak (2θ)
Before	7.146	42.8
After	6.779	43.6

**Table 8:** X-ray diffraction data before and after fatigue testing.

Fatigue Test	FWHM	Peak (2θ)
Before	6.518	44.1
After	6.758	44.04
